# Effect of supplementation with flaxseed oil and different doses of fish oil for 2 weeks on plasma phosphatidylcholine fatty acids in young women

**DOI:** 10.1038/s41430-018-0174-2

**Published:** 2018-05-30

**Authors:** Leanne Hodson, Francesca L. Crowe, Kirsten J. McLachlan, C. Murray Skeaff

**Affiliations:** 1 Department of Human Nutrition, University of Otago, Dunedin, New Zealand; 2 Present Address: Oxford Centre for Diabetes, Endocrinology and Metabolism (OCDEM), University of Oxford, Churchill Hospital, Oxford, OX3 7LE UK; 3 Present Address: Institute of Applied Health Research, University of Birmingham, Edgbaston, Birmingham B15 2TT UK

## Abstract

**Background/objectives:**

Although assumed, it remains unclear that fatty acid (FA) biomarkers of n-3 long-chain PUFA reflect wide ranges of intake. However, to be utilised as biomarkers, to predict dietary intake, dose–response curves that cover a spectrum of intakes are required. The aim of the study was to investigate whether the FA composition of plasma phosphatidylcholine (PC) is a sensitive biomarker of n-3 FAs from fish oil, across a range of supplementation doses, and alpha-linolenic acid (ALA) supplementation, in young, healthy women.

**Subjects/methods:**

A total of 303 young women were randomised to intakes ranging between 0.33 and 4.50 g EPA+DHA/day from fish oil (not all doses used in each year) or flaxseed oil (5.90–6.60 g/d) daily for 14 days in a series of trials, over 5 years. Fasting blood was collected at baseline (day 0) and day 14 and plasma PC FA composition, total and HDL-cholesterol and triglyceride concentrations measured.

**Results:**

Fourteen days supplementation with fish oil significantly (*P* < 0.01) increased, in a dose-dependent fashion, plasma PC EPA, DPA and DHA at all doses except 1 and 3 mL/day. For the combined group of women who consumed any fish oil there was a 16% (*P* < 0.01) decrease in plasma triacylglycerol concentrations after 14 days supplementation. Flaxseed oil supplementation for 14 day resulted in significant (*P* < 0.01) increases in ALA, EPA and DPA, whilst DHA remained unchanged.

**Conclusion:**

Our data demonstrate plasma PC is a sensitive biomarker of n-3 FA intake and reflects changes within 14 days across a range of intakes.

## Introduction

Plasma, erythrocyte and platelet phospholipids are the blood lipid fractions most abundant in n-3 long-chain polyunsaturated fatty acids (LCPUFA). Dose–response studies, typically with only three ‘distinct’ levels of n-3 fatty acid (FA), have shown blood phospholipids reflect changes in n-3 LCPUFA intake in a dose–response manner [1–3]. However, for FA to be utilised as biomarkers to predict dietary intake, dose–response curves that cover a spectrum of FA intake are required and although assumed, it remains unclear that FA biomarkers of n-3 LCPUFA reflect a wide range of intakes. Another important source of n-3 LCPUFA could come from the precursor alpha-linolenic acid (ALA). Longer-term studies have found the abundance of ALA, EPA and docosapentaenoic acid (DPA) increased in FA biomarkers with an increase in ALA intake with the data for DHA being less clear [4–11].

Although evidence from randomised controlled trials has not proven n-3 LCPUFA lowers cardiovascular disease (CVD) risk [12], intervention studies have clearly demonstrated n-3 LCPUFA (as fish oil or ethyl esters of EPA and DHA) have a triglyceride-lowering effect [13]. A large proportion of these studies have been undertaken in middle-aged adults [13, 14]; it remains unclear whether n-3 LCPUFA have a similar effect in young adults.

We sought to investigate the effect of short-term (14 days) supplementation with EPA and DHA (given as fish oil), across a range of doses, on plasma phosphatidylcholine (PC) n-3 FA levels and plasma lipid concentrations, along with the effect of short-term (14 days) ALA supplementation on plasma PC n-3 FA status in young, healthy women.

## Subjects and methods

### Participants

Participants were recruited from an undergraduate nutrition course at the University of Otago and were eligible if they were 18 years or older and were not allergic to fish or nuts. Ethical approval was given for the study from The Human Ethics Committee, University of Otago. All participants gave informed written consent after receiving both a verbal and written explanation of the study. The experiment was part of an undergraduate teaching and learning activity that was conducted annually for five years from 2003 to 2007.

### Study design and supplementation

Participants were randomised to receive fish oil or flaxseed oil in capsule form and consumed the allocated daily dose, with food for 14 days. Participants were asked to maintain their usual diet and physical activity; if they consumed fish they were instructed to continue their usual pattern of consumption. MaxEPA (Seven Seas Health Company UK) fish oil capsules were used in 2003 and 2004 and Omega-3 Salmon Oil capsules (Thompson’s, Auckland, New Zealand) from 2005 to 2007; flaxseed oil was from Waihi Bush (Geraldine, New Zealand). Manufacturer’s information indicated that each capsule of MaxEPA contained 190 mg of EPA and 110 mg of DHA, each capsule of Omega-3 Salmon Oil contained 180 mg of EPA and 120 mg of DHA, and each capsule of flaxseed oil contained 600 mg of ALA. The doses of fish oil used in the trials were 1, 2, 3, 4, 5, 6, 10 and 15 capsules/day, not all doses were used in each year (due to participant numbers), whereas the dose of flaxseed oil was always 10 capsules/day. Intakes (g/d) of EPA and DHA and ALA were estimated on the basis of manufacturers’ information about the FA content of the fish oil and flaxseed oil and the number of capsules assigned to be consumed (Supplementary Table 1). Compliance was assessed in all years by change in FA composition of plasma PC and in 2005, 2006 and 2007 (but not 2003 and 2004) compliance was also assessed by a daily diary of capsule consumption.

### Biochemical and lipid analysis

On days 0 and 14 venous blood samples were collected from participants after an overnight fast and plasma isolated and stored [15]. Plasma total, high-density lipoprotein (HDL) cholesterol and triglyceride concentrations were measured and plasma low-density lipoprotein (LDL) cholesterol concentrations calculated [16]. The analytical coefficient of variation for the measurement of total cholesterol was less than 3% for plasma total cholesterol, HDL-cholesterol and triglyceride.

Plasma lipids were extracted, after the addition of a known amount of an internal standard (diheptadecanoyl [17:0] PC), according to the method of Bligh and Dyer [17]. Plasma PC was separated using thin-layer chromatography as we have previously described for erythrocyte PC [18] and PC FAs converted to FA methyl esters (FAMEs). Separation and quantitation of the plasma PC FAMEs was achieved using a DB-225 megabore column (25 m × 0.53 mm internal diameter; film thickness 0.25 µm; J & W Scientific) installed on an HP-6890 Series Gas Chromatograph (GC) with flame ionisation detection [15, 19]. Students performed the lipid extraction and thin-layer chromatography, under supervision, whereas a qualified research technician performed the GC analysis of samples. Plasma FA were recorded as molecular percentages (mol%), defined as the number of molecules of the individual FA as a percentage of the total number of FA molecules. The concentrations (µmol/L) of FAs were calculated based on the area of the internal standard (diheptadecanoyl [17:0] PC) peak.

### Statistical analysis

Data were analysed using the statistical package STATA (version 11). Statistical differences in the plasma lipids and FA composition of PC between day 0 and day 14 were determined using a paired *t*-test. All comparisons were two-sided and changes were considered statistically significant when the *P*-value was less than 0.01; this value was chosen to reduce the chances of concluding erroneously that a difference existed between days 14 and 0.

As subjects consumed EPA and DHA (given as fish oil), across a range of doses (Supplementary Table 1) over a 14-day period, this provided the opportunity to determine if there was a dose–response effect. The change from day 0 to 14 in plasma PC n-3 FA or plasma lipid concentration was calculated for each participant and these values used in the regression analysis to test for a dose–response effect. The dose–response relation between the increase in EPA intake and change in EPA composition of plasma PC was estimated using regression analysis, adjusting for year of the study and baseline (i.e., day 0) FA composition. The dose–response relation was expressed as the incremental change (95% CI) in mol% per g/d or µmol/L per g/d increase in intake of EPA. A simple plot of the results suggested a curvilinear relation between increasing intake of EPA and change in mol% and µmol/L EPA in plasma PC; therefore, a quadratic term was included in the model. The same procedure was used to explore the dose–response relation between DHA intake and DHA composition of plasma PC; however, a quadratic term was dropped from the model because it was not statistically significant (*P* = 0.150). A similar approach was used to calculate the dose response relation between EPA+DHA intake and plasma lipid concentrations (mmol/L), with the dose response expressed as the incremental change (95% CI) per g/d increase of EPA+DHA.

## Results

### Baseline characteristics

Three hundred and three women participated in the supplementation trials between 2003 and 2007, of which we have complete data for 294. The participants’ (*n* = 294) were aged 22.1 years (4.0) (mean (SD)) with a BMI of 22.6 kg/m^2^ (2.9); mean plasma lipid concentrations were 4.45 (0.80) mmol/L for plasma total cholesterol, 2.51 (0.68) mmol/L for plasma LDL-cholesterol, 1.51 (0.35) mmol/L for plasma HDL cholesterol, and 1.11 (0.40) mmol/L for plasma triglycerides. The characteristics of women assigned to the fish or flaxseed oil groups did not differ. The number of women enrolled each year and randomised to the different fish oil groups or the flaxseed oil group is shown in Supplementary Table 1. Ninety-four percent of participants from 2005 through 2007 completed a daily diary of capsule consumption; self-reported compliance indicated that 97% of the assigned capsules were consumed.

### Effect of fish oil supplementation on plasma PC n-3 FA

**Fig. 1:**
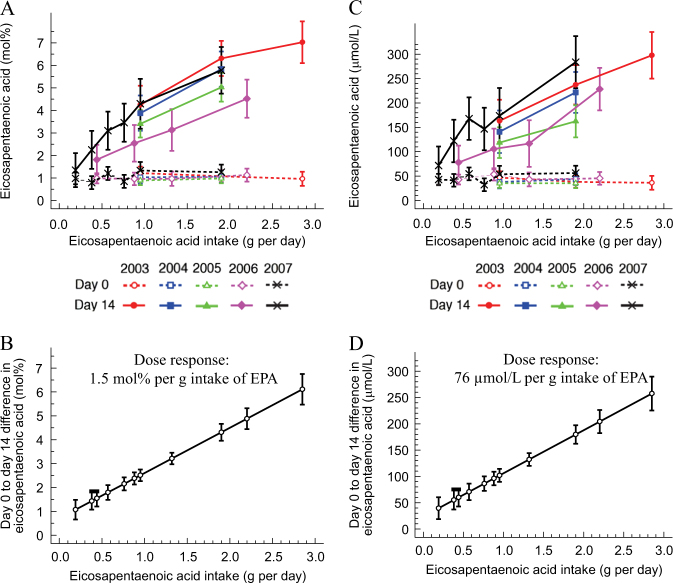
Effect of fish oil supplementation on **a** the molecular percent (mol %) and **b** concentration (µmol/L) of EPA in plasma phosphatidylcholine (PC). Values are unadjusted means (95% CI) of the change, from days 0 to 14, in the mol % and µmol/L of EPA in plasma PC and are shown by dose of EPA intake and by year in which the study was conducted. The point estimates occur at the particular doses used in each year. Values are predicted means (95% CI) calculated by regressing the dose of EPA on the change, from days 0 to 14, in the mol % and µmol/L of EPA in plasma PC with adjustment for year of study and baseline EPA composition of plasma PC. The point estimates shown on the graph were calculated for each dose of EPA intake (g/d) on the **c** mol % and **d** µmol/L

Daily supplementation with different amounts of fish oil (EPA+DHA) significantly (*P* < 0.01) increased plasma PC EPA mol% and µmol/L at all doses of fish oil intakes except the one mL dose (Tables 1 and 2). The mean increase, unadjusted analysis, in EPA mol% and µmol/L between days 0 and 14 by dose of EPA is shown in Fig. 1a, c; the increase was dose-dependent and did not differ by year of study (*P* = 0.988, interaction between dose and year). According to this association, the dose response, over 14 days, in plasma PC EPA was 1.5 mol% or 76 µmol/L per g EPA (Fig. 1b, d). Notably, daily supplementation with fish oil significantly increased the abundance (as mol% and µmol/L) of DPA in plasma PC, in most, but not all doses of fish oil (Tables 1 and 2).

**Table 1:** Long-chain n-3 polyunsaturated fatty acid composition (mol%) of plasma phosphatidylcholine at before and after 14 days of consuming the oil supplement

Intake (g/d)			*n*	Eicosapentaenoic acid (µmol/L)			Docosapentaenoic acid (µmol/L)			Docosahexaenoic acid (µmol/L)		
EPA	DHA	EPA+DHA		Day 0^a^	Day 14^a^	Difference^b^	Day 0^a^	Day 14^a^	Difference^b^	Day 0^a^	Day 14^a^	Difference^b^
0.19	0.14	0.33	15	1.0 (0.5)	1.3 (0.3)	0.4 (−0.1, 0.8)	0.7 (0.2)	0.8 (0.2)	0.1 (−0.1, 0.3)	3.0 (1.0)	3.3 (0.9)	0.3 (−0.6, 1.2)
0.38	0.28	0.66	12	0.8 (0.2)	2.2 (0.7)	1.5 (0.7, 2.2)^c^	0.6 (0.2)	0.9 (0.3)	0.3 (0.2, 0.5)^c^	2.7 (0.8)	3.7 (0.8)	1.0 (0.5, 1.5)^c^
0.44	0.28	0.72	19	1.0 (0.4)	1.6 (0.6)	0.8 (0.1, 1.5)^c^	0.7 (0.2)	0.9 (0.3)	0.2 (0.0, 0.4)^c^	3.6 (1.2)	3.6 (1.0)	0.0 (−0.8, 0.9)
0.55	0.42	0.99	12	1.2 (1.0)	3.1 (1.1)	1.9 (1.1, 2.6)^c^	0.7 (0.3)	1.0 (0.4)	0.3 (0.1, 0.5)^c^	3.1 (1.4)	4.3 (1.3)	1.2 (0.2, 2.2)^c^
0.76	0.56	1.32	12	0.8 (0.2)	3.5 (0.7)	2.6 (2.1, 3.2)^c^	0.7 (0.2)	1.1 (0.2)	0.4 (0.3, 0.6)^c^	3.1 (0.8)	4.5 (0.9)	1.4 (0.8, 2.0)^c^
0.88	0.56	1.44	13	1.0 (0.3)	2.5 (1.0)	1.6 (0.8, 2.4)^c^	0.7 (0.2)	1.1 (0.3)	0.3 (0.1, 0.5)^c^	3.4 (0.9)	4.6 (1.1)	1.2 (−0.2, 2.6)
0.95	0.55	1.50	47	1.0 (0.5)	3.7 (1.3)	2.7 (2.3, 3.2)^c^	0.8 (0.3)	1.2 (0.3)	0.4 (0.3, 0.5)^c^	3.4 (1.1)	4.6 (1.2)	1.3 (0.9, 1.7)^c^
0.95	0.70	1.65	7	1.3 (1.0)	4.3 (0.7)	3.0 (1.2, 4.7)^c^	0.7 (0.2)	1.1 (0.3)	0.3 (0.0, 0.7)^c^	3.4 (0.7)	4.7 (1.1)	1.3 (0.0, 2.6)^c^
1.32	0.84	2.16	10	1.0 (0.6)	3.1 (1.5)	2.2 (0.5, 3.8)^c^	0.8 (0.2)	1.1 (0.3)	0.3 (0.1, 0.6)^c^	3.3 (0.7)	4.4 (1.4)	1.1 (−0.7, 2.9)
1.90	1.10	3.00	48	1.0 (0.5)	5.6 (2.0)	4.6 (3.8, 5.4)^c^	0.8 (0.2)	1.4 (0.3)	0.6 (0.5, 0.7)^c^	3.5 (1.0)	5.2 (1.3)	1.8 (1.3, 2.2)^c^
1.90	1.40	3.30	8	1.3 (0.7)	5.8 (2.0)	4.5 (2.0, 7.0)^c^	1.0 (0.2)	1.6 (0.3)	0.6 (0.1, 1.0)^c^	4.1 (1.1)	5.6 (1.5)	1.6 (−0.2, 3.4)
2.20	1.40	3.60	12	1.1 (0.5)	4.5 (2.3)	3.4 (1.3, 5.5)^c^	0.8 (0.3)	1.4 (0.3)	0.6 (0.2, 0.9)^c^	3.3 (0.8)	5.3 (1.2)	1.9 (1.3, 2.6)^c^
2.85	1.65	4.50	10	1.0 (0.4)	7.0 (2.5)	6.1 (3.4, 8.7)^c^	0.7 (0.3)	1.7 (0.4)	1.0 (0.5, 1.5)^c^	3.7 (1.1)	6.4 (1.0)	2.7 (1.4, 4.1)^c^

^a^ Values are mean (SD)

^b^ Values are mean (99% CI)

^c^ Day 14 significantly different from day 0, paired *t*-test *P* < 0.01

**Table 2:** Long-chain n-3 polyunsaturated fatty acid composition (µmol/L) of plasma phosphatidylcholine at before and after 14 days of consuming the oil supplement

Intake (g/d)			*n*	Eicosapentaenoic acid (µmol/L)			Docosapentaenoic acid (µmol/L)			Docosahexaenoic acid (µmol/L)		
EPA	DHA	EPA+DHA		Day 0^a^	Day 14^a^	Difference^b^	Day 0^c^	Day 14^a^	Difference^b^	Day 0^a^	Day 14^a^	Difference^b^
0.19	0.14	0.33	15	43 (19)	72 (51)	29 (−15, 72)	31 (10)	43 (32)	12 (−11, 34)	144 (92)	180 (167)	37 (−89, 163)
0.38	0.28	0.66	12	41 (10)	122 (55)	81 (37, 125)^c^	29 (8)	49 (24)	20 (3, 37)^c^	151 (75)	198 (68)	47 (−2, 96)
0.44	0.28	0.72	19	42 (22)	78 (45)	36 (6, 65)^c^	31 (15)	45 (24)	13 (0, 27)^c^	157 (92)	177 (105)	20 (−61, 101)
0.55	0.42	0.99	12	54 (36)	168 (79)	113 (62, 164)^c^	35 (20)	53 (24)	18 (5, 32)^c^	147 (80)	227 (88)	80 (28, 132)^c^
0.76	0.56	1.32	1	32 (11)	147 (67)	115 (58, 171)^c^	27 (8)	48 (20)	21 (4, 39)^c^	121 (42)	193 (90)	72 (−3, 148)
0.88	0.56	1.44	13	53 (29)	106 (43)	52 (10, 94)^c^	39 (19)	43 (9)	3 (−13, 19)	201 (131)	186 (30)	−15 (−123, 93)
0.95	0.55	1.50	47	39 (23)	136 (60)	97 (75, 119)^c^	29 (14)	42 (19)	13 (7, 19)^c^	126 (57)	168 (75)	42 (17, 66)^c^
0.95	0.70	1.65	7	54 (38)	174 (40)	120 (66, 173)^c^	29 (9)	42 (9)	12 (3, 22)^c^	141 (38)	184 (32)	43 (14, 72)^c^
1.32	0.84	2.16	10	43 (36)	117 (37)	74 (17, 130)^c^	32 (11)	42 (12)	10 (−3, 23)	148 (71)	176 (65)	28 (−73, 129)
1.90	1.10	3.00	48	38 (18)	200 (88)	162 (128, 196)^c^	29 (12)	49 (19)	21 (13, 28)^c^	129 (50)	185 (70)	56 (36, 76)^c^
1.90	1.40	3.30	8	56 (32)	284 (124)	228 (88, 367)^c^	42 (8)	74 (29)	32 (2, 62)^c^	175 (60)	274 (114)	98 (−16, 213)
2.20	1.40	3.60	12	45 (23)	229 (145)	183 (61, 305)^c^	32 (12)	72 (32)	40 (14, 66)^c^	133 (44)	282 (135)	149 (48, 250)^c^
2.85	1.65	4.50	10	36 (15)	298 (138)	262 (121, 403)^c^	28 (11)	74 (31)	46 (15, 77)^c^	143 (62)	272 (106)	129 (37, 221)^c^

^a^ Values are mean (SD)

^b^ Values are mean (99% CI)

^c^ Day 14 significantly different from day 0, paired *t*-test *P* < 0.01

**Fig. 2:**
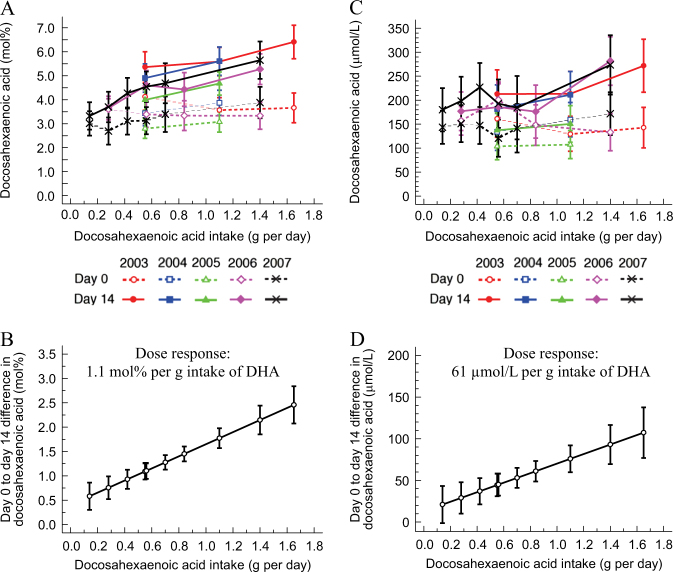
Effect of fish oil supplementation on **a** the molecular percent (mol %) and **b** concentration (µmol/L) of DHA in plasma phosphatidylcholine (PC). Values are unadjusted means (95% CI) of the change, from days 0 to 14, in the mol % and µmol/L of DHA in plasma PC and are shown by dose of DHA intake and by year in which the study was conducted. The point estimates occur at the particular doses used in each year. Values are predicted means (95%CI) calculated by regressing the dose of DHA on the change, from days 0 to 14, in the mol % and µmol/L of DHA in plasma PC with adjustment for year of study and baseline DHA composition of plasma PC. The point estimates shown on the graph were calculated for each dose of DHA intake (g/d) on the **c** mol % and **d** µmol/L

Daily supplementation with fish oil also significantly (*P* < 0.01) increased plasma PC DPA and DHA mol% at all doses of fish oil intake except one and three mL (Table 1) but changes occurred at fewer fish oil doses when expressed as a concentration (µmol/L) (Table 2). The mean increase, unadjusted analysis, in DHA mol% and µmol/L between day 0 and 14 by dose of fish oil intake is shown in Fig. 2a,c; the increase was linearly dose-dependent and did not differ by year of study (*P* = 0.524, interaction between dose and year). The predicted mean (95%CI) increase, over 14 days, in plasma PC DHA mol% and µmol/L across the range of daily supplemental DHA intake is shown in Fig. 2b,d. According to this association, the dose response, over 14 days, in plasma PC DHA was 1.1 mol% or 61 µmol/L per g intake of DHA (Fig. 2b,d).

### Effect of flaxseed oil supplementation on plasma PC n-3 FA

Daily supplementation with flaxseed oil for 14 days increased plasma PC ALA by 0.7 mol% (95% CI, 0.6–0.9; *P* < 0.001) or 32 µmol/L (95% CI, 14–46, *P* < 0.001) (Table 3). The proportions and concentrations of plasma PC FA as EPA and DPA were significantly (*P* < 0.01) increased, with no change in DHA, after 14 days (Table 3). The changes in FA composition with flaxseed oil consumption did not differ by year in which the study was conducted (*P* > 0.6).

**Table 3:** N-3 polyunsaturated fatty acid composition of plasma phosphatidylcholine before and after 14 days of consuming flaxseed oil (10 mL/day)

	Mol%			µmol/L		
Fatty acid	Day 0^a^	Day 14^a^	Difference^b^	Day 0^a^	Day 14^a^	Difference^b^
18:3n-3	0.4 (0.2)	1.1 (0.5)	0.7 (0.6, 0.9)^c^	14 (7)	46 (31)	32 (23, 42)^c^
20:5n-3	1.0 (0.4)	2.0 (1.3)	1.0 (0.7, 1.4)^c^	39 (19)	89 (82)	49 (24, 75)^c^
22:5n-3	0.8 (0.2)	1.0 (0.3)	0.2 (0.1, 0.3)^c^	30 (13)	42 (33)	12 (2, 23)^d^
22:6n-3	3.3 (1.0)	3.4 (1.4)	0.1 (−0.4, 0.5)	132 (51)	148 (122)	16 (−24, 56)

^a^ Values are mean (SD), *n* = 69

^b^ Values are mean (99% CI), *n* = 69

^c^ Day 14 significantly different from day 0, paired *t*-test *P* < 0.001

^d^ Day 14 significantly different from day 0, paired *t*-test *P* < 0.01

### Effect of fish oil and flaxseed oil supplementation on plasma lipids

Daily fish oil supplementation for 14 days did not significantly alter plasma total cholesterol concentrations within any one group of fish oil intake (Table 4); however, when the results for all participants who received fish oil were combined (*n* = 234), mean plasma total and LDL cholesterol concentrations were significantly (*P* < 0.001) higher at day 14 compared with day 0. For this combined group, mean intake of fish oil, weighted for the proportion of participants in each dose group, was 6.1 mL/d, or 1.8 g of EPA+DHA (Table 4). Plasma HDL-cholesterol concentrations were unaltered by fish oil consumption (Table 4). Mean plasma triglyceride concentrations were significantly (*P* < 0.01) lowered by consuming 1.50, 1.65, 3.00 and 3.60 g EPA+DHA/day for 14 day; they were also significantly lower (by 15%, *P* < 0.001 day 14 vs day 0) when results were combined for all fish oil consumers (Table 4).

**Table 4:** Plasma lipid concentrations before and after 14 days of consuming the oil supplement

Intake (g/d)			*n*	Total cholesterol (mmol/L)			LDL cholesterol (mmol/L)			HDL cholesterol (mmol/L)			Triacylglycerol (mmol/L)		
EPA	DHA	EPA+DHA		Day 0^a^	Day 14^a^	Difference^b^	Day 0^a^	Day 14^a^	Difference^b^	Day 0^a^	Day 14^a^	Difference^b^	Day 0^a^	Day 14^a^	Difference^b^
0.19	0.14	0.33	15	4.29 (0.52)	4.40 (0.61)	0.11 (−0.11, 0.33)	2.50 (0.51)	2.59 (0.59)	0.10 (−0.13, 0.32)	1.40 (0.20)	1.37 (0.24)	−0.02 (−0.14, 0.10)	0.86 (0.17)	0.95 (0.29)	0.08 (−0.06, 0.23)
0.38	0.28	0.66	12	5.15 (0.90)	5.35 (1.09)	0.20 (−0.41, 0.80)	2.74 (0.74)	3.01 (1.01)	0.27 (−0.37, 0.91)	1.74 (0.36)	1.77 (0.33)	0.03 (−0.13, 0.19)	1.48 (0.43)	1.25 (0.51)	−0.23 (−0.80, 0.35)
0.44	0.28	0.72	19	4.20 (0.66)	4.31 (0.75)	0.11 (−0.12, 0.34)	2.56 (0.54)	2.72 (0.59)	0.16 (−0.09, 0.41)	1.47 (0.33)	1.42 (0.32)	−0.05 (−0.19, 0.10)	1.07 (0.34)	1.05 (0.32)	−0.01 (−0.17, 0.14)
0.55	0.42	0.99	12	4.15 (0.77)	4.41 (0.93)	0.26 (−0.11, 0.63)	2.27 (0.67)	2.58 (0.81)	0.31 (−0.06, 0.69)	1.42 (0.26)	1.47 (0.27)	0.04 (−0.16, 0.25)	1.02 (0.30)	0.80 (0.20)	−0.22 (−0.56, 0.13)
0.76	0.56	1.32	12	4.25 (0.39)	4.51 (0.67)	0.26 (−0.26, 0.77)	2.34 (0.39)	2.64 (0.63)	0.30 (−0.15, 0.76)	1.48 (0.35)	1.46 (0.35)	−0.02 (−0.25, 0.21)	0.96 (0.20)	0.91 (0.36)	−0.05 (−0.42, 0.32)
0.88	0.56	1.44	13	4.64 (0.85)	4.55 (0.92)	−0.09 (−0.51, 0.33)	2.74 (0.91)	2.67 (0.86)	−0.07 (−0.48, 0.35)	1.73 (0.38)	1.74 (0.44)	0.00 (−0.14, 0.15)	1.06 (0.28)	0.92 (0.27)	−0.14 (−0.32, 0.03)
0.95	0.55	1.50	47	4.50 (0.92)	4.61 (0.84)	0.11 (−0.08, 0.30)	2.36 (0.61)	2.59 (0.63)	0.23 (0.04, 0.42)	1.51 (0.35)	1.58 (0.35)	0.06 (−0.03, 0.16)	1.20 (0.51)	1.00 (0.44)	−0.20 (−0.33, −0.07)^c^
0.95	0.70	1.65	7	3.81 (0.73)	4.09 (0.90)	0.28 (−0.20, 0.77)	2.14 (0.58)	2.44 (0.82)	0.30 (−0.16, 0.75)	1.19 (0.37)	1.31 (0.32)	0.12 (−0.06, 0.29)	1.04 (0.40)	0.75 (0.41)	−0.29 (−0.43, −0.16)^c^
1.32	0.84	2.16	10	4.64 (0.80)	4.39 (0.80)	−0.25 (−0.74, 0.24)	3.08 (0.68)	2.85 (0.68)	−0.23 (−0.73, 0.27)	1.35 (0.43)	1.39 (0.39)	0.03 (−0.14, 0.21)	1.27 (0.39)	0.93 (0.30)	−0.34 (−0.71, 0.03)
1.90	1.10	3.00	48	4.67 (0.90)	4.73 (0.87)	0.06 (−0.12, 0.24)	2.62 (0.81)	2.81 (0.83)	0.19 (−0.03, 0.41)	1.51 (0.34)	1.54 (0.38)	0.03 (−0.07, 0.13)	1.15 (0.33)	0.91 (0.37)	−0.24 (−0.38, −0.10)^c^
1.90	1.40	3.30	8	4.07 (0.48)	4.45 (0.57)	0.38 (−0.29, 1.04)	2.12 (0.29)	2.50 (0.37)	0.37 (−0.07, 0.82)	1.49 (0.42)	1.60 (0.48)	0.12 (−0.12, 0.35)	0.99 (0.28)	0.75 (0.24)	−0.25 (−0.49, −0.01)
2.20	1.40	3.60	12	4.29 (0.39)	4.34 (0.52)	0.05 (−0.32, 0.42)	2.46 (0.47)	2.59 (0.50)	0.13 (−0.23, 0.48)	1.65 (0.39)	1.61 (0.35)	−0.04 (−0.24, 0.17)	1.07 (0.25)	0.83 (0.23)	−0.23 (−0.37, −0.10)^c^
2.85	1.65	4.50	10	4.39 (0.78)	4.82 (0.92)	0.43 (−0.04, 0.91)							1.38 (0.50)	1.27 (0.53)	−0.11 (−0.62, 0.40)
All doses of fish oil^d^			225	4.46 (0.81)	4.58 (0.84)	0.12 (0.04, 0.20)^c^	2.51 (0.67)	2.68 (0.71)	0.17 (0.09, 0.26)^c^	1.51 (0.36)	1.53 (0.36)	0.02 (−0.02, 0.06)	1.13 (0.39)	0.96 (0.38)	−0.17 (−0.24, −0.11)^c^
Flaxseed oil															
10			69	4.44 (0.73)	4.49 (0.88)	0.05 (−0.06, 0.16)	2.55 (0.68)	2.63 (0.73)	0.08 (−0.02, 0.17)	1.50 (0.30)	1.50 (0.32)	0.00 (−0.04, 0.04)	1.03 (0.36)	0.96 (0.35)	−0.07 (−0.14, −0.01)

^a^ Mean difference (99% CI) between days 14 and 0 for all doses of fish oil

^b^ Paired *t*-test day 14 compared with day 0

^c^ Day 14 significantly different from day 0, paired *t*-test *P* < 0.01

^d^ Results for combined group of all participants consuming fish oil

There was no significant dose-response relation between total n-3 LCPUFA intake (i.e., EPA+DHA) and total cholesterol (*P* = 0.235), LDL-cholesterol (*P* = 0.955), or HDL-cholesterol (*P* = 0.440) concentrations (Table 4) or with plasma triglyceride concentrations. Consuming 10 mL of flaxseed oil, containing 6 g of ALA, daily for 14 days did not significantly alter plasma lipid concentrations (Table 4).

## Discussion

As the usefulness of plasma PC as a biomarker of n-3 FA intake has not been extensively examined, we assessed changes in plasma PC n-3 FAs before and after supplementation with fish oil (across a range of doses) or supplementation with ALA. Our results clearly demonstrate the FA composition of plasma PC to be a sensitive biomarker of n-3 LCPUFA intake, with small increases in EPA and DHA intake (from fish oil) being reflected within 14 days, in a dose-dependent fashion. The increase in EPA and DHA abundance with increasing dose of fish oil, and the increase in ALA abundance with flaxseed oil supplementation indicate a high degree of compliance over the 14-day period. EPA and DPA, two FA formed by the metabolic interconversion of ALA, were also increased with flaxseed oil supplementation, whilst DHA content remained unchanged.

Blood lipid n-3 FAs have been extensively utilised as biomarkers of n-3 intakes as they are predominantly derived from dietary intake [1, 20]. This relationship was reported by Lands et al. [21] who described an empirical association between the maintenance of plasma phospholipid LCPUFA and the dietary intakes of n-6 and n-3 FAs. More recently, Patterson et al. [3] undertook a dose–response study and systematic review to investigate the relationship between diet and blood n-3 FA. They found the abundance of whole blood, erythrocyte and plasma phospholipid EPA+DHA to increase in a linear manner with dietary intakes up to 1 g/d EPA+DHA. In the present study, we found plasma PC EPA and DHA to increase in a linear manner with intakes up to 4.5 g/d EPA+DHA. As the participants in the present study consumed fish oil supplements for 14 days, the generalisability of our estimates of the dose–response relation between intake of n-3 LCPUFA and change in abundance of plasma PC EPA or DHA depend on whether this period is sufficient for maximum changes in FA composition to occur. Browning et al. [2] estimated the time to maximal change in plasma PC EPA content ranged from 5 to 18 days depending on the dose, with the majority of change occurring in the first few days of supplementation. Thus, our estimate of the dose–response for EPA is unlikely to be underestimated to a significant degree. In contrast, Browning et al. [2] found the time to peak changes in plasma PC DHA composition were longer, ranging between 12 and 32 days, which is in-line with others [22]. Thus, it is most likely our estimate for dose–response for DHA underestimates maximum incorporation.

Our findings of significant increases in plasma PC ALA, EPA and DPA, with no change in DHA after 14 days supplementation with flaxseed oil are in line with previous work [4–10]. Although, Hennebelle et al. [11] reported ALA supplementation for 4 weeks increased total plasma EPA and DHA abundance in older (73y) but not younger (25y) adults. The synthesis of ALA to EPA, DPA and DHA occurs primarily in liver endoplasmic reticulum where there is competition between n-6 and n-3 FA for the same elongase and desaturase enzymes [23]. The conversion of ALA may be downregulated by increased availability of conversion products; consumption of a fish—compared to a beef-based diet decreased conversion of DPA to DHA [24]. Additional factors that may influence the synthesis of DHA from ALA include smoking tobacco [25], alcohol consumption [26] and hormonal status [27].

Numerous studies have demonstrated the hypo-triglyceridaemic effect of n-3 LCPUFA (from fish or supplements) [13, 14]. The significant decrease in plasma triglyceride concentrations in the present study was unexpected, not least as supplementation was short-term (14 days) and participants were young, healthy females with relatively low baseline plasma triglyceride concentrations. In contrast, Flock et al. [28] reported no change in plasma triglyceride concentrations in healthy, young individuals consuming up to 1.80 g of EPA plus DHA/day for 5 months. The discrepancy in findings between studies may be partly due to the higher doses of EPA plus DHA in the present study. Supplementation with ALA did not decrease plasma triglyceride concentrations, consistent with some [4] but not all [7] previous reports.

Evidence for changes in plasma total, LDL- and HDL-cholesterol concentrations after n-3 LCPUFA supplementation are less consistent, although it appears increases in LDL-cholesterol are more marked in individuals with hyper-triglyceridaemia [13, 14]. We found a small but significant increase in plasma total cholesterol, which can be explained by an increase in LDL-cholesterol after n-3 LCPUFA supplementation, with no effect of ALA supplementation as previously reported [14, 29]. A proposed mechanism for the increase in LDL-cholesterol concentrations is via an increased conversion rate of VLDL to LDL [29].

Our study has some limitations. We used MaxEPA for two and salmon oil for three of the trials; however, as there were only small compositional differences between the oils, we combined the dose of fish oil but used actual intake of supplemental EPA and DHA in the statistical calculations of the dose-response relationships. Our study participants were young, healthy females who were undergraduate students studying nutrition. We did not assess participants’ background diet or lifestyle habits at baseline or over the course of the study. Although they were instructed to make no changes other than taking the oil supplements, it is possible that alterations to diet and lifestyle occurred during the course of the study. Nor did we control for hormone status/phase of menstrual cycle or use of oral contraceptives. As participants undertook a component of the analytical work, on their own samples, and although clear instructions were provided and they were supervised, the variation between participants and across years is likely to be higher than if the analytical work had been undertaken by more experienced technicians. Finally, we only studied young, healthy females so we can only speculate that the dose–response incorporation of EPA and DHA into plasma PC would be similar in young, healthy men. This seems likely, given the results of a population survey reporting men and women (25–44 years) had similar-3 LCPUFA status [30].

Taken together, our data describe, the dose-response relationship between EPA and DHA intake and plasma PC EPA and DHA mol% and µmol/L after 14 days of supplementation, in young healthy women. We found that per g intake per day increase in EPA results in a 1.5 mol% or 76 µmol/L increase in plasma PC EPA abundance whilst per g intake per day increase in DHA results in an increase of 1.1 mol% or 61 µmol/L in plasma PC DHA abundance. These data highlight that even modest changes in dietary fat intake are reflected rapidly by plasma PC n-3 FA. In long-term studies where n-3 FA biomarkers are only measured at the study beginning and end to determine compliance, it may prove difficult to distinguish true compliers from non-compliers if measuring plasma lipid pools [15, 31, 32]; however, the proportion of erythrocyte DHA has been reported to characterise adherence to EPA and DHA intakes in long-term interventions [31]. Finally, our data highlight that short-term supplementation with ALA is reflected rapidly by plasma PC and has only a modest, if any effect on n-3 LCPUFA status in this cohort.

## References

[1] Hodson L, Skeaff CM, Fielding BA (2008). Fatty acid composition of adipose tissue and blood in humans and its use as a biomarker of dietary intake. Prog Lipid Res.

[2] Browning LM, Walker CG, Mander AP, West AL, Madden J, Gambell JM (2012). Incorporation of eicosapentaenoic and docosahexaenoic acids into lipid pools when given as supplements providing doses equivalent to typical intakes of oily fish. Am J Clin Nutr.

[3] Patterson AC, Chalil A, Aristizabal Henao JJ, Streit IT, Stark KD (2015). Omega-3 polyunsaturated fatty acid blood biomarkers increase linearly in men and women after tightly controlled intakes of 0.25, 0.5, and 1 g/d of EPA+DHA. Nutr Res.

[4] Barcelo-Coblijn G, Murphy EJ, Othman R, Moghadasian MH, Kashour T, Friel JK (2008). Flaxseed oil and fish-oil capsule consumption alters human red blood cell n-3 fatty acid composition: a multiple-dosing trial comparing 2 sources of n-3 fatty acid. Am J Clin Nutr.

[5] Goyens PL, Spilker ME, Zock PL, Katan MB, Mensink RP (2006). Conversion of alpha-linolenic acid in humans is influenced by the absolute amounts of alpha-linolenic acid and linoleic acid in the diet and not by their ratio. Am J Clin Nutr.

[6] Mantzioris E, James MJ, Gibson RA, Cleland LG (1994). Dietary substitution with an alpha-linolenic acid-rich vegetable oil increases eicosapentaenoic acid concentrations in tissues. Am J Clin Nutr.

[7] Schwab US, Callaway JC, Erkkila AT, Gynther J, Uusitupa MI, Jarvinen T (2006). Effects of hempseed and flaxseed oils on the profile of serum lipids, serum total and lipoprotein lipid concentrations and haemostatic factors. Eur J Nutr.

[8] Wallace FA, Miles EA, Calder PC (2003). Comparison of the effects of linseed oil and different doses of fish oil on mononuclear cell function in healthy human subjects. Br J Nutr.

[9] Wilkinson P, Leach C, Ah-Sing EE, Hussain N, Miller GJ, Millward DJ (2005). Influence of alpha-linolenic acid and fish-oil on markers of cardiovascular risk in subjects with an atherogenic lipoprotein phenotype. Atherosclerosis.

[10] Zhao G, Etherton TD, Martin KR, West SG, Gillies PJ, Kris-Etherton PM (2004). Dietary alpha-linolenic acid reduces inflammatory and lipid cardiovascular risk factors in hypercholesterolemic men and women. J Nutr.

[11] Hennebelle M, Courchesne-Loyer A, St-Pierre V, Vandenberghe C, Castellano CA, Fortier M (2016). Preliminary evaluation of a differential effect of an alpha-linolenate-rich supplement on ketogenesis and plasma omega-3 fatty acids in young and older adults. Nutrition.

[12] Rizos EC, Ntzani EE, Bika E, Kostapanos MS, Elisaf MS (2012). Association between omega-3 fatty acid supplementation and risk of major cardiovascular disease events: a systematic review and meta-analysis. JAMA.

[13] Harris WS (1997). n-3 fatty’ acids and serum lipoproteins: human studies. Am J Clin Nutr.

[14] Balk EM, Lichtenstein AH, Chung M, Kupelnick B, Chew P, Lau J (2006). Effects of omega-3 fatty acids on serum markers of cardiovascular disease risk: a systematic review. Atherosclerosis.

[15] Hodson L, Eyles HC, McLachlan KJ, Bell ML, Green TJ, Skeaff CM (2014). Plasma and erythrocyte fatty acids reflect intakes of saturated and n-6 PUFA within a similar time frame. J Nutr.

[16] Hodson L, Skeaff CM, Chisholm WA (2001). The effect of replacing dietary saturated fat with polyunsaturated or monounsaturated fat on plasma lipids in free-living young adults. Eur J Clin Nutr.

[17] Bligh DG, Dyer WJ (1959). A rapid method of total lipid extraction and purification. Can J Biochem Physiol.

[18] Hodson L, Skeaff CM, Wallace AJ, Arribas GL (2002). Stability of plasma and erythrocyte fatty acid composition during cold storage. Clin Chim Acta.

[19] Holub BJ, Skeaff CM (1987). Nutritional regulation of cellular phosphatidylinositol. Methods Enzymol.

[20] Serra-Majem L, Nissensohn M, Overby NC, Fekete K (2012). Dietary methods and biomarkers of omega 3 fatty acids: a systematic review. Br J Nutr.

[21] Lands WE, Libelt B, Morris A, Kramer NC, Prewitt TE, Bowen P (1992). Maintenance of lower proportions of (n - 6) eicosanoid precursors in phospholipids of human plasma in response to added dietary (n - 3) fatty acids. Biochim Biophys Acta.

[22] Katan MB, Deslypere JP, van Birgelen AP, Penders M, Zegwaard M (1997). Kinetics of the incorporation of dietary fatty acids into serum cholesteryl esters, erythrocyte membranes, and adipose tissue: an 18-month controlled study. J Lipid Res.

[23] Arterburn LM, Hall EB, Oken H (2006). Distribution, interconversion, and dose response of n-3 fatty acids in humans. Am J Clin Nutr.

[24] Pawlosky R, Hibbeln J, Lin Y, Salem N (2003). n-3 fatty acid metabolism in women. Br J Nutr.

[25] Pawlosky RJ, Hibbeln JR, Salem N (2007). Compartmental analyses of plasma n-3 essential fatty acids among male and female smokers and nonsmokers. J Lipid Res.

[26] Pawlosky RJ, Hibbeln JR, Herion D, Kleiner DE, Salem N (2009). Compartmental analysis of plasma and liver n-3 essential fatty acids in alcohol-dependent men during withdrawal. J Lipid Res.

[27] Giltay EJ, Gooren LJ, Toorians AW, Katan MB, Zock PL (2004). Docosahexaenoic acid concentrations are higher in women than in men because of estrogenic effects. Am J Clin Nutr.

[28] Flock MR, Skulas-Ray AC, Harris WS, Etherton TD, Fleming JA, Kris-Etherton PM (2013). Determinants of erythrocyte omega-3 fatty acid content in response to fish oil supplementation: a dose-response randomized controlled trial. J Am Heart Assoc.

[29] Barcelo-Coblijn G, Murphy EJ (2009). Alpha-linolenic acid and its conversion to longer chain n-3 fatty acids: benefits for human health and a role in maintaining tissue n-3 fatty acid levels. Prog Lipid Res.

[30] Crowe FL, Skeaff CM, Green TJ, Gray AR (2008). Serum n-3 long-chain PUFA differ by sex and age in a population-based survey of New Zealand adolescents and adults. Br J Nutr.

[31] Patterson AC, Metherel AH, Hanning RM, Stark KD (2014). The percentage of DHA in erythrocytes can detect non-adherence to advice to increase EPA and DHA intakes. Br J Nutr.

[32] Skeaff CM, Hodson L, McKenzie JE (2006). Dietary-induced changes in fatty acid composition of human plasma, platelet, and erythrocyte lipids follow a similar time course. J Nutr.

